# Multipath Lightweight Deep Network Using Randomly Selected Dilated Convolution

**DOI:** 10.3390/s21237862

**Published:** 2021-11-26

**Authors:** Sangun Park, Dong Eui Chang

**Affiliations:** School of Electrical Engineering, Korea Advanced Institute of Science and Technology, Daejeon 34141, Korea; undol26@kaist.ac.kr

**Keywords:** lightweight deep network, object classification, network design

## Abstract

Robot vision is an essential research field that enables machines to perform various tasks by classifying/detecting/segmenting objects as humans do. The classification accuracy of machine learning algorithms already exceeds that of a well-trained human, and the results are rather saturated. Hence, in recent years, many studies have been conducted in the direction of reducing the weight of the model and applying it to mobile devices. For this purpose, we propose a multipath lightweight deep network using randomly selected dilated convolutions. The proposed network consists of two sets of multipath networks (minimum 2, maximum 8), where the output feature maps of one path are concatenated with the input feature maps of the other path so that the features are reusable and abundant. We also replace the 3×3 standard convolution of each path with a randomly selected dilated convolution, which has the effect of increasing the receptive field. The proposed network lowers the number of floating point operations (FLOPs) and parameters by more than 50% and the classification error by 0.8% as compared to the state-of-the-art. We show that the proposed network is efficient.

## 1. Introduction

Object detection is one of the essential techniques that robots need to perform a variety of tasks. While humans can easily find and identify objects, robots are unable to do so. However, it is technically challenging to detect objects quickly and accurately in robot vision. Owing to its high importance, this field has received increased attention in recent years.

Deep convolutional neural networks (DCNNs) have attracted extensive attention in various computer vision applications such as object detection [1,2,3,4,5,6,7], object classification [8,9,10,11,12,13,14,15], and image segmentation [16,17,18,19,20]. DCNNs are composed of a series of convolutional layers, resulting in abundant features, more parameters, and complicated structures. These properties lead to a significant improvement in performance. Some of the prominent research involving DCNNs is as follows: combining networks in networks (NIN [21]), reducing the number of parameters by proposing a bottleneck layer (GoogLeNet [22]), placing several simple networks (VGGNet [11]), connecting an extra path between different layers (ResNet [12]), concatenating from previous layers to the next layers (DenseNet [13]), and increasing the number of channels as the layers get deeper (PyramidNet [23]).

However, as the applications of deep learning networks become more complex, the size of the model has increased rapidly. Nevertheless, deep learning networks are being deployed to lightweight devices such as mobile devices and automobiles. Large models have the following risks: memory limits, training/inference speed, performance degradation, and dead channels. The gradient required for training is proportional to the size of the model, so, even if the learning speed is increased through distributed learning, the training takes more time as the model grows. Many existing studies have attempted to solve this problem through training with multiple graphics processing units (GPUs), such as data parallelization [24,25,26,27] and model parallelism [28,29,30]. Moreover, there are unnecessary channels that have little effect on the output result during the learning process. They can be a significant waste that continuously increases the computational complexity of the model. The pruning method [31] can resolve this problem.

Lightweight model design is designing a model with fewer parameters and computations while maintaining a similar level of performance. If the amount of computations is reduced, it enables deployment of the DCNN on low-power devices and secures real-time performance, and if the number of parameters is reduced, resources required for model storage and transmission are reduced. Therefore, it is very valuable to conduct research on lightweight model design.

In order to design lightweight models, we consider the following two fundamental questions:

1. Can the network be designed in a different way to make the model lighter?
2. How can we obtain richer feature maps than state-of-the-art (SOTA) traditional DCNNs?

We answer these questions with our proposed network, called the “multipath lightweight deep network using randomly selected dilated convolution”. It consists of at least two multipath networks and uses a randomly selected dilated convolution to expand the receptive field.

Our main contributions are as follows.

- First, we design an extensible and modular network architecture. This model can be plugged into any existing network.
- Second, we reduce the the number of floating point operations (FLOPs) and parameters by more than 50%. Our model is composed of multipath network structures, so it is optimized for parallelization, and the model is light because the computation loss is small.
- Third, the proposed model has a wide receptive field and fewer parameters than the existing ones. This model can be placed in front of any network using the 3×3 standard convolution.

The remainder of this paper is organized as follows. Section 2 presents the related works about object classification methods and lightweight DCNN architectures. Section 3 explains and analyzes our proposed model. Section 4 shows our experimental results. Section 5 discusses the effect of our proposed network. Section 6 concludes the paper and presents possible future work.

## 2. Related Work

### 2.1. Object Classification

AlexNet [9] was the first network to popularize convolutional neural networks (CNNs). Unlike LeNet [8], AlexNet placed convolutional layers one after the other and improved performance by learning deep networks (8 layers) while utilizing GPU and rectified linear unit (ReLU) functions. The full-scale deep-layer era started with GoogLeNet [22]. GoogLeNet implemented an inception design to obtain features of different scales by applying different-scale convolution filters to the same layer. Especially, the bottleneck layer has a great effect on dimensionality reduction and computational cost reduction, so that a deeper network (22 layers) can be learned. However, as the network gets deeper, the gradient value saturates, which makes learning extremely slow. In addition, the error increases as the number of parameters increases. VGGNet [11] improved the performance by learning a deep network (19 layers) with only the simplest 3×3 convolution without changing the size of the receptive field.

The residual network (ResNet) [12] solved the vanishing gradient problem by adding shortcuts between adjacent layers, optimizing very deep networks (152 layers), and obtaining better performance with increasing depth based on the uncomplicated network, VGGNet. Later, DenseNet [13] showed improved performance with fewer parameters as compared to ResNet. Unlike ResNet, DenseNet connected a layer to all previous layers via shortcut paths. By stacking feature maps, DenseNet can obtain very abundant feature maps and reduce the vanishing-gradient problems. Dual path networks (DPNs) [32] combined the advantages of ResNet for feature reuse and DenseNet for exploring new features. As the name suggests, DPNs consist of dual paths. One path is a ResNet network, while the other path is a DenseNet network.

### 2.2. Lightweight CNN Architectures

Existing object classification and detection models require a lot of computation power for training and testing, so expensive equipment such as GPUs is necessary. The model size is also relatively large, and it takes a lot of time to train and test, so improvement in model size and computational efficiency is essential for real-time application. To solve this problem, various attempts have been made to compress the deep network or reduce the amount of computation. In particular, recent studies have explored reducing the weight of the model while maintaining the performance of the existing model.

Based on the effect of Inception, Xception [33] proposed a depthwise-separable convolution network. SqueezeNet [34] reduced computation cost and the number of input channels by replacing some 3×3 convolution layers with point-wise convolution. MobileNet [35] proposed a lightweight architecture structure that can run on mobile devices through depthwise-separable convolutions. ShuffleNet [36] proposed a more efficient structure than MobileNet by applying group convolution to bottleneck layer operation and shuffle channels.

CondenseNet [14] achieved similar accuracy with a lower computational cost than other lightweight models such as MobileNet and ShuffleNet. It is a model with similar accuracy to DenseNet requiring one tenth of the computation power by pruning connections with less feature reuse by using learned group convolution, and increasing the growth rate as the network gets deeper. MobileNetV2 [37] proposed linear bottlenecks and an inverted residual to upgrade the architecture while improving performance in all indicators such as accuracy, the number of parameters, and amount of computation. ShuffleNetv2 [38] added a channel-splitting module to input and used concatenation instead of addition, resulting in faster processing speed with similar accuracy to ShuffleNetv1 as well as MobileNetV2. MobileNetv3 [39] used the NASNet [40] architecture to explore the structure and improved the performance by modifying the searched structure. CondenseNetV2 [15] used reactivating obsolete features not considered in CondenseNet and ShuffleNetV2. In addition, by adding a sparse feature reactivation (SFR) module after the existing learned group convolution, features were concatenated after processing.

## 3. Methods

In this section, we introduce the details of our proposed network. The model we refer to as the basic structure is DenseNet-based (including CondenseNet and CondenseNetV2) because the information of the previous layer is concatenated and the features are reused. That is, it is characterized by having much richer features compared to other networks that do not concatenate. In particular, CondenseNet can be used for lightweight models because it reduces the number of parameters ten times as compared to DenseNet and provides similar performance. Hence, we chose this as the basic model. Since CondenseNet has recently been improved to CondenseNetV2, we also applied the proposed method to CondenseNetV2.

The major differences between the proposed network and other network architectures are the presence of multipath networks and the expansion of the receptive field. Dividing the path into a pair of cross-shaped paths is the key to reducing the number of FLOPs and parameters. A detailed description is provided in Section 3.2 and Section 3.3.

**Figure 1:**
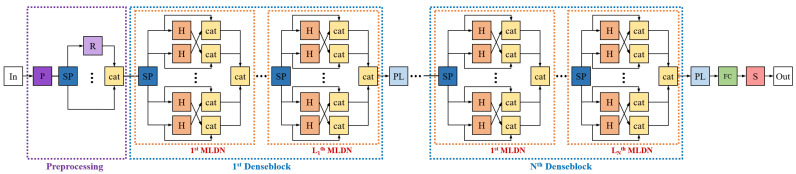
Overall procedure. The input goes through preprocessing and several dense blocks and pooling layers. During this procedure, the feature maps reduce the the number of FLOPs and parameters.

Figure 1 shows the overall architecture of the proposed network. MLDN described in Section 3.2 represents our proposed multipath lightweight deep network. In the figure, P in the dark purple box is the preprocessing module, R in the light purple box is ResNet, SP in the dark blue box splits the input into the number of paths, cat in the yellow box is concatenation, H in the orange box is the composite function of MLDN, PL in the light blue box is the average pooling layer, FC in the green box denotes fully connected layers, and S in the red box is a softmax function. In and out in the white box are the input images and the predicted class, respectively. This network predicts the classes of objects through preprocessing, dense blocks, transition layers (average pooling), fully connected layers, and a softmax function. Preprocessing helps generate diverse feature maps. A denseblock has *L* MLDN and MLDN has *p* multiple paths. The various feature maps are produced after passing through each MLDN and denseblock. The pooling layer reduces the size of the output channels of each denseblock.

### 3.1. Preprocessing

**Figure 2:**
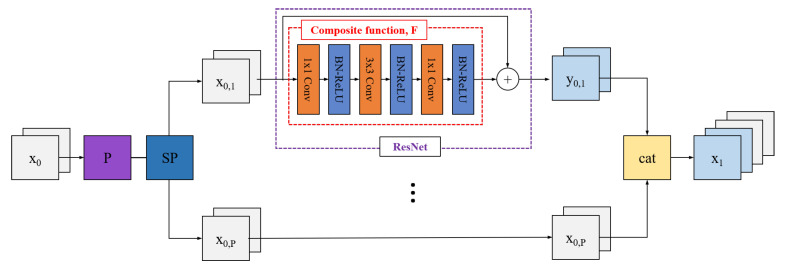
Preprocessing. Before splitting input feature maps, we apply initial convolution. Only one path has the ResNet module, and the other paths just pass the input feature maps.

Figure 2 describes preprocessing. Before using the input image itself, we first increment the feature maps by passing an initial convolutional layer (dark purple box). We mentioned that the network is divided into multiple paths. ResNet-18, which has the least number of FLOPs among various ResNets, is applied once to one of these paths. Henceforth, we use ResNet-18 wherever we mention ResNet.

This is similar to applying network-in-network (NIN) on GoogLeNet. Since it is one of the many processes, it does not increase the number of FLOPs or parameters significantly. Since ResNet performs an element-wise sum by shortcut connection of input features to output features, it transforms the feature itself without using it. Therefore, the path passing through ResNet and the path not passing through ResNet can be configured with different feature maps to improve performance.

The composite function, F(), consists of combination of convolution, batch normalization (BN) [41], and ReLU [42] layers including the bottleneck layer. We adjust the feature maps so that the number of output feature maps that pass through ResNet equals the number of output feature maps on the paths that do not. We can express the ResNet path as $y_{1}=x_{1}⊕F\left(x_{1}\right)$, where $x_{1}$ are the input feature maps of the ResNet path, $y_{1}$ are the output feature maps of ResNet, and ⊕ is the element-wise sum operation.

### 3.2. Multipath Lightweight Deep Network

Most deep networks except GoogLeNet and DPN have only one path. GoogLeNet separates the network by placing the network in the network, but the networks do not exchange feature information with each other. DPN combines two networks to take advantage of both networks.

We propose a lightweight deep network with multiple paths to make a model suitable for weight reduction. The key to making a lightweight model as compared to existing networks is by reducing the number of parameters through multipath networks. Depending on the network design, the number of paths can be defined as a divisor of the growth rate. The growth rate of [13] controls the amount of information added to the network at each layer. For example, if the growth rate is 8, the possible paths are 2, 4, and 8. Algorithm 1 describes the proposed network, multipath lightweight deep network (MLDN), in *n*th denseblock in CondenseNet.
**Algorithm 1** MLDN in *n*th denseblock in CondenseNet**Input**: $x_{n}$**for**l=1 to $L_{n}$ **do**    $x_{n,l,2p-1},x_{n,l,2p}=\mathrm{split}\left(x_{n,l}\right)$ where 1≤p≤P/2    **for** p=1 to P/2 **do**        $y_{n,l,2p-1}=\mathrm{cat}\left(x_{n,l,2p-1},H_{n,l,2p}\left(x_{n,l,2p}\right)\right)$        $y_{n,l,2p}=\mathrm{cat}\left(x_{n,l,2p},H_{n,l,2p-1}\left(x_{n,l,2p-1}\right)\right)$    **end for**    $x_{n,l+1}=\mathrm{cat}\left(y_{n,l,1},y_{n,l,2},\ldots ,y_{n,l,2p-1},y_{n,l,2p}\right)$**end for****Output**: $x_{n+1}=x_{n,L_{n}+1}$

Here, *n* is the index of the denseblock, *l* is the index of layers, $L_{n}$ is the number of layers in the *n*th denseblock, *p* is the index of the path, *P* is the number of paths, H(·) is the composite function, cat is the concatenation operation, and split is the split operation.

**Figure 3:**
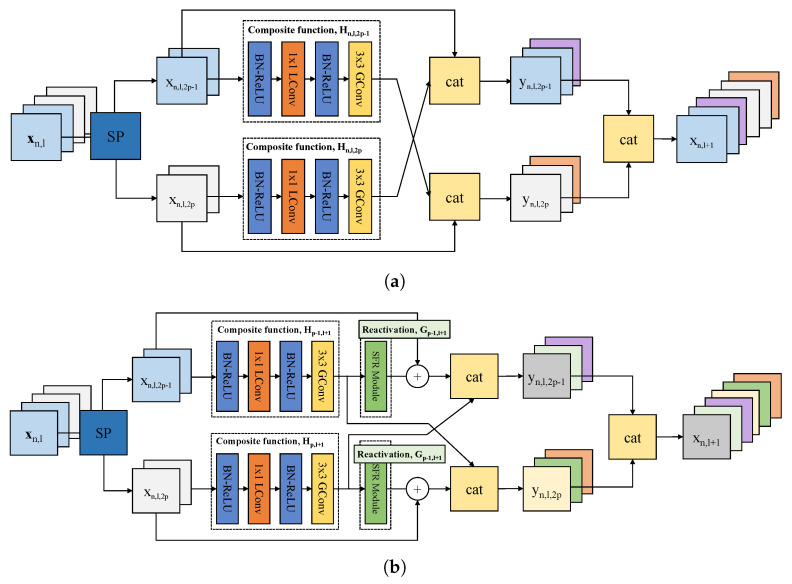
Multipath lightweight deep network (MLDN) in *l*th layers, *n*th denseblock. (**a**) MLDN in CondenseNet; (**b**) MLDN in CondenseNetV2. They pass their output feature maps to the other path.

Figure 3 is an example of dividing the path into two (*P* = 2) in the *l*th layer. In Figure 3a, when *l* = 1, MLDN is explained as follows. First, input feature maps ($x_{n,1}$) split by the number of 2 ($x_{n,1,1},x_{n,1,2}$). These split input feature maps are passed through the composite function ($H_{n,1,1}\left(x_{n,1,1}\right),H_{n,1,2}\left(x_{n,1,2}\right)$). Next, they concatenate with the input feature maps of the opposite path ($y_{n,1,1}=\mathrm{cat}\left(x_{n,1,1},H_{n,1,2}\left(x_{n,1,2}\right)\right),y_{n,1,2}=\mathrm{cat}\left(x_{n,1,2},H_{n,1,1}\left(x_{n,1,1}\right)\right)$). Finally, all output feature maps are re-concatenated ($x_{n,2}=\mathrm{cat}(y_{n,1,1},y_{n,1,2}$). These output feature maps become the next input feature maps for the (l+1)th layer. This is repeated on all layers. In this way, information is exchanged between paths. Each composite function has two iterations of BN, ReLU, and convolution maps in series. For the first convolutional layer, learned group convolution (L-Conv) removes unimportant connections. For the second convolutional layer, group convolution (G-Conv) reduces the computational cost by partitioning the input features. In the composite function process, the number of feature maps does not increase and a constant *k*, the number of feature maps, is generated because of a bottleneck layer. More information about the composite function is in [14].

CondenseNetV2 adds a sparse feature reactivation (SFR) module after the composite function, and the output feature maps of this SFR module are added by an element-wise sum with the input feature maps. Finally, the input feature maps from the (2p−1)th path and output feature maps from the 2pth path are concatenated as in CondenseNet. Algorithm 2 explains how to plug in MLDN to CondenseNetV2. The composite function *G* in the SFR increases the output channels equal to the input feature maps. The rest is the same notation as in Algorithm 1. Since it is similar to MLDN+CondenseNet, the description has been omitted.
**Algorithm 2** MLDN in *n*th denseblock in CondenseNetV2**Input**: $x_{n}$**for**l=1 to $L_{n}$ **do**    $x_{n,l,2p-1},x_{n,l,2p}=\mathrm{split}\left(x_{n,l}\right)$ where 1≤p≤P/2    **for** p=1 to P/2 **do**        $y_{n,l,2p-1}=\mathrm{cat}\left(x_{n,l,2p-1}⊕G_{n,l,2p-1}\left(H_{n,l,2p-1}\left(x_{n,l,2p-1}\right)\right),H_{n,l,2p}\left(x_{n,l,2p}\right)\right)$        $y_{n,l,2p}=\mathrm{cat}\left(x_{n,l,2p}⊕G_{n,l,2p}\left(H_{n,l,2p}\left(x_{n,l,2p}\right)\right),H_{n,l,2p-1}\left(x_{n,l,2p-1}\right)\right)$    **end for**    $x_{n,l+1}=\mathrm{cat}\left(y_{n,l,1},y_{n,l,2},\ldots ,y_{n,l,2p-1},y_{n,l,2p}\right)$**end for****Output**: $x_{n+1}=x_{n,L_{n}+1}$

Our network architectures are shown in Table 1. A denseblock is composed of L-Conv (learned convolution) and G-Conv (grouped convolution). We choose L=14 layers and *P* = [2,4,8] for each denseblock. The total growth rate *k* is [8,16,32], so the growth rate per path $k_{p}$ is [4,4,4] ($k_{n,p}=k_{n}/p_{n}$). In the last layer, the output of the FC layer is the same as the number of labels in the dataset. As an example, the outputs of the FC layer in the cases of the CIFAR-10 and CIFAR-100 datasets are 10 and 100, respectively.

### 3.3. Randomly Selected Dilated Convolution

A large receptive field can be used to improve network performance. However, this considerably increases the number of parameters and risks overfitting. Therefore, in general DCNNs, these problems are solved by combining convolution and pooling to lower the cost. Factorized convolution reduces the number of parameters and deepens the layers by replacing the feature maps of a large receptive field with a few other small feature maps, but it increases the depth of the network. To widen the receptive field and not deepen the network at the same time, we draw inspiration from dilated convolutions [43].

Dilated convolution expands small feature maps (3×3) into large feature maps (5×5, 7×7 …) but, conversely, we reduce large feature maps to small feature maps while using all the information in the large feature maps. We propose a randomly selected dilated convolution (RSDC) with an extended receptive field but a relatively shallow layer.

**Figure 4:**
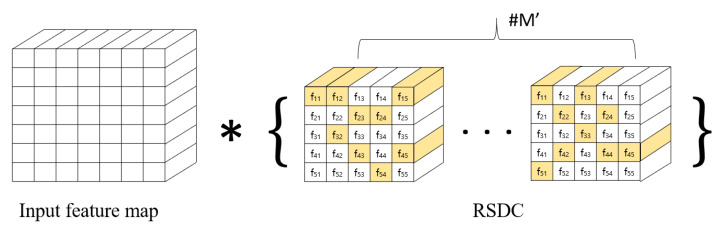
Randomly selected dilated convolution (RSDC) when kernel size is 5. The yellow on the right of the figure denotes randomly selected weights. There are $M^{\prime }$ output feature maps.

Figure 4 explains our RSDC when the kernel size of the RSDC is 5. The left side of the figure shows the input feature maps, * is the convolution symbol, and the right side of the figure shows the RSDC, where RSDC consists of $M^{\prime }$ feature maps. In this case, there are 25 weights. If we use this value as it is, however, it is the same as increasing the receptive field. We randomly select 9 of these weights and perform convolution with them. The reason for choosing 9 weights is that the size of the existing standard convolution is 3×3.

Algorithm 3 explains our network with RSDC in a general case. The $f^{\;\mathrm{input}}$ denotes the RSDC feature maps depicted on the right side of Figure 4 and $f^{\;\mathrm{output}}$ denotes randomly selected weights shown in yellow on the right side of Figure 4. The height and width of the input feature maps of RSDC are denoted by *h*, *w*, respectively; $k_{rsdc}$ is the predefined kernel size for RSDC, e.g., 5 or 7; and *i*, *j* are height and width indices of the output feature maps of RSDC, respectively. The rand(*x*) function picks a random value of *x*, and the append(*x*) function appends *x* to the output.
**Algorithm 3** RSDC before applying 3×3 standard convolution feature maps**Input**: $f^{\;\mathrm{input}}=\left\{f_{h,w}\right\},\;\mathrm{where}\;1\leq h,w\leq k_{rsdc}$**for**i=1 to 3 **do**    **for** j=1 to 3 **do**        $f=\mathrm{rand}(f_{h,w})$        **while** *f* in $f_{i,j}$ **do**           $f=\mathrm{rand}(f_{h,w})$        **end while**        $f_{i,j}=\mathrm{append}\left(f\right)$    **end for****end for****Output**: $f^{\;\mathrm{output}}=\left\{f_{i,j}\right\},\;\mathrm{where}\;1\leq i,j\leq 3$

In general, according to the factorized convolution method, the number of *M* feature maps of size $k_{rsdc}$ that can be factorized into several 3×3 feature maps is given by:

$$k_{rsdc}^{2}\times M\to 3^{2}\times M\times \frac{\left(k_{rsdc}-1\right)}{2}.$$

Since our RSDC uses only 9 weights, we can think of it as using 3×3 convolution feature maps (i.e., $k_{rsdc}=3$ in the preceding equation). Therefore, to use fewer feature maps as compared to existing feature maps while obtaining a large receptive field, the following should be satisfied:

$$\begin{array}{c} M^{\prime }<M\times \frac{\left(k_{rsdc}-1\right)}{2}, \end{array}$$

where $M^{\prime }$ is the number of feature maps of RSDC in Figure 4.

The MLDN exceeds SOTA (shown in Section 4.3.2), so we apply RSDC to MLDN. Among the composite functions of MLDN, a 3×3 convolution exists only once at the end. The proposed RSDC is located instead of the 3×3 standard convolution of MLDN. In one path of the first denseblock, we have $L_{1}$ layers and growth rate $k_{p}$, so the increasing number of output feature maps is $L_{1}\times k_{p}$. Since the growth rate is too small in the first denseblock, we apply RSDC from the second denseblock.

## 4. Experimental Results

### 4.1. Datasets

We evaluated our proposed network on the CIFAR-10 and CIFAR-100 [44] datasets, and the ImageNet (ILSVRC) [45] datasets. The CIFAR-10 and CIFAR-100 datasets are composed of 32×32 pixel-sized RGB images corresponding to 10 and 100 classes, respectively. They have 50,000 training images and 10,000 testing images. We used a standard data-augmentation method [21,46,47,48,49,50] where the images were zero-padded to 4 pixels on all sides with a probability of 0.5, randomly cropped, and mirrored horizontally to keep the size of 32×32 pixels. We separated the 10,000 images from the training dataset into the validation dataset. The ImageNet dataset consists of 1000 classes and contains a total of 1.2 million training images and 50,000 validation images. We adopted the data-augmentation method of [12] at training time, rescaled the input image to 256×256 at test time, and then performed a 224×224 center crop.

### 4.2. Training Settings

All models were trained by stochastic gradient descent (SGD) using similar optimization hyperparameters as in [14,15]. We adopted the Nesterov momentum weight of 0.9 without dampening and used a weight decay of 1 × 10^−4^. All models were trained with a mini-batch size of 32 for 300 epochs. The cosine-shaped learning rate [51] was used, and it started at 0.1 and gradually decreased to 0. Dropout [52,53] with a drop rate of 0.1 was applied to train.

### 4.3. Performance Evaluation

#### 4.3.1. The Effect of ResNet

**Figure 5:**
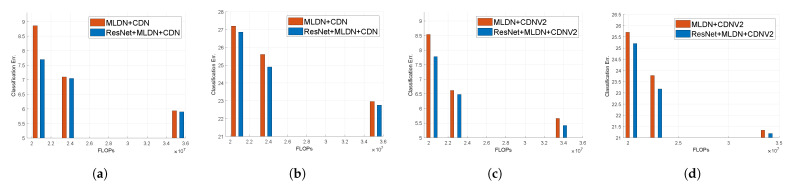
Comparison of the classification error rates with respect to the number of FLOPs on the effect of ResNet and MLDN. (**a**) MLDN in CondenseNet on CIFAR-10. (**b**) MLDN in CondenseNet on CIFAR-100. (**c**) MLDN in CondenseNetV2 on CIFAR-10. (**d**) MLDN in CondenseNetV2 on CIFAR-100.

We now show experimental validation for the fact that using ResNet is more effective than not using it as explained in Section 3.1. Figure 5 shows the classification error as a function of the number of FLOPs. In this paper, the classification error means top-1 error. Detailed values are given in Table 2.

Although the number of FLOPs in the case of the network with ResNet is slightly larger than those without ResNet (about 1M), we see a decrease in the classification error of the network. These values can be seen in the 1st (CDN) to the 3rd (MLDN+CDN) rows of the first column (Model) in Table 2. In the case of CondensetNetV2, a similar effect can be seen as shown in the 4th row (CDNV2) to the 6th row (MLDN+CDNV2) of the first column (Model) of Table 2.

#### 4.3.2. The Effect of Multiple Paths

We studied the effect of changing the number of paths and the growth rate on the proposed network. We designed the network such that each denseblock has 14 layers. We chose three sets of paths: *P* = [2,2,2], *P* = [2,4,4], and *P* = [2,4,8], such that *P* = [2,4,8] implies that the first denseblock has 2 paths, the second denseblock has 4 paths, and the third denseblock has 8 paths. The growth rate of the paths also increases such that the first denseblock has growth rate 8, the second denseblock has growth rate 16, and the third denseblock has growth rate 32. Figure 5 depicts the effect of multiple paths. In the case of MLDN+CDN/CDNV2 or ResNet+MLDN+CDN/CDNV2, *p* decreases from left (smaller FLOPs) to right (larger FLOPs) such as *P* = [2,2,2], *P* = [2,4,4], and *P* = [2,4,8]. Larger paths reduce the number of FLOPs because more operations are processed at one time. However, the number of output feature maps (actually, $\frac{\mathrm{\#output}\;\mathrm{feature}\;\mathrm{maps}}{\mathrm{\#paths}}$) used for training becomes smaller and the classification error increases.

Detailed values are given in Table 2. It can be seen that the best result is shown when *p* = [2,2,2] with a constant size. Our MLDN+CDN model shows a 0.13% and 0.95% improvement in the classification error on CIFAR-10 and CIFAR-100, respectively. It reduces the number of FLOPs and parameters by 54.1% and 53.8% compared to the CDN, respectively. MLDN+CDNV2 improves by 0.37% and 1.12% in the classification error on CIFAR-10 and CIFAR-100 respectively. It reduces the number of FLOPs and parameters by 53% and 51% compared to the CDNV2, respectively.

#### 4.3.3. The Effect of Changing MLDN Hyperparameters

**Figure 6:**
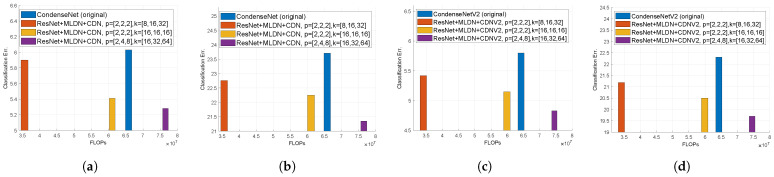
Comparison of the classification error rates with respect to the number of FLOPs using MLDN on the effect of changing hyperparameters. (**a**) MLDN in CondenseNet on CIFAR-10. (**b**) MLDN in CondenseNet on CIFAR-100. (**c**) MLDN in CondenseNetV2 on CIFAR-10. (**d**) MLDN in CondenseNetV2 on CIFAR-100.

We also performed the experiment with various MLDN hyperparameters. We compare the original network, our best models, increased and constant growth rates, and doubling growth rates in Figure 6.

First, we experimented with constant growth rates such as *k* = [16,16,16], and *k* = [32,32,32] when *p* = [2,2,2], so the growth rates per path were $k_{p}$ = [8,8,8] and $k_{p}$ = [16,16,16], respectively, which are sufficient to train well. However, in the case of *k* = [32,32,32], the number of FLOPs is too large to be meaningful. Hence, we did not plot for *k* = [32,32,32] in Figure 6 due to the scale problem. We see that as the growth rates increase with constant values, the classification error decreases but the number of FLOPs increases. There are trade-offs between these two.

Second, we experimented with doubling the growth rates. Unlike the best results where we chose *p* = [2,2,2] and *k* = [8,16,32], the number of FLOPs is slightly larger than the original (11M), but the classification error is reduced by 0.7% when *p* = [2,4,8] and *k* = [16,32,64]. This is because doubling *k* is enough to satisfy the training well. When *p* = [2,2,2] and *k* = [16,32,64], the number of FLOPs becomes too large to be worthwhile. Except for *p* = [2,4,8] and *k* = [16,32,64], the rest are not depicted in Figure 6 because of scale issues.

#### 4.3.4. The Effect of RSDC

**Figure 7:**
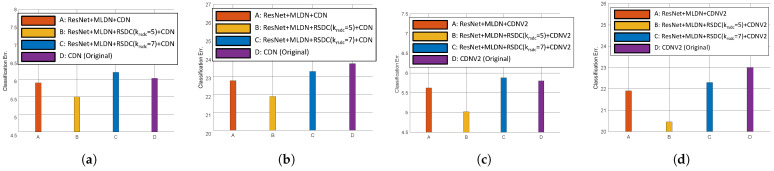
Comparison of the classification error rates with respect to the number of FLOPs using RSDC. (**a**) ResNet+ MLDN+RSDC+CDN on CIFAR-10. (**b**) ResNet+MLDN+RSDC+CDN on CIFAR-100. (**c**) ResNet+MLDN+RSDC+CDNV2 on CIFAR-10. (**d**) ResNet+MLDN+RSDC+CDNV2 on CIFAR-100.

From the above results (in Section 4.3.1 and Section 4.3.2), we confirm that having multiple paths and ResNet is more effective than the existing networks. Therefore, we applied RSDC to MLDN with ResNet to experiment with its effectiveness. The results were best when *p* = [2,2,2] and *k* = [8,16,32], so we set the same for this experiment. Figure 7 compares original, MLDN, and RSDC.

First, we experimented by changing the kernel size of RSDC, $k_{rsdc}$. The classification error was lowest when $k_{rsdc}$ was 5. This is because the input image size of the CIFAR dataset is so small (32×32) and it is inefficient to use a large receptive field such as $k_{rsdc}$ = 7. In addition, $k_{rsdc}=5$ has larger selected weights than $k_{rsdc}$ (9/25 vs. 9/49) for the same number of output feature maps. Detailed values are given in Table 3.

**Figure 8:**
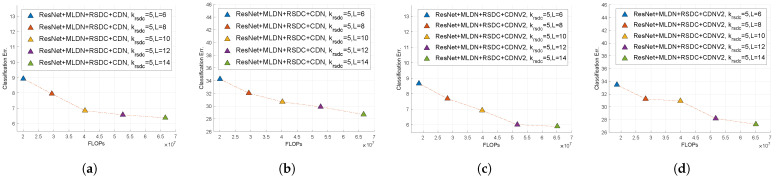
Comparison of the classification error rates with respect to the number of FLOPs using RSDC with changes in the number of layers when $k_{rsdc}$ = 5 and *G* = *C* = 2. (**a**) ResNet+MLDN+RSDC+CDN on CIFAR-10. (**b**) ResNet+MLDN+RSDC+ CDN on CIFAR-100. (**c**) ResNet+MLDN+RSDC+CDNV2 on CIFAR-10. (**d**) ResNet+MLDN+RSDC+CDNV2 on CIFAR-100.

Second, we changed the number of layers. In [14,15], the condensation factor (*C*) and the number of groups (*G*) is 4. The condensation factor is the removal rate of the filter weight. With this factor, it is impossible to experiment with varying reductions in *L* for computational reasons such as size mismatch. Therefore, we changed this factor to 2. We changed *L* by 2 from 6 to 14. The smallest number of FLOPs and parameters is when *L* = 6, and the largest number of FLOPs and parameters is when *L* = 14. The classification error at *L* = 10 in Figure 8a or *L* = 12 in Figure 8c,d is similar to the previous classification error, but since the number of FLOPs is reduced by about 15 million, it is the best choice to obtain the lowest classification error with respect to the the number of FLOPs. When the number of layers is smaller than the *L* mentioned above, the classification error tends to increase remarkably because $M^{\prime }$, the number of output feature maps of RSDC, is not sufficient.

#### 4.3.5. Comparison with SOTA

Table 4 compares the classification error rates with SOTA for various networks on the CIFAR datasets. It can be seen that our proposed network (bottom row of Table 4) significantly reduces the number of FLOPs and parameters compared to SOTA. In particular, compared to CondenseNet, the number of FLOPs is reduced by about 55%, and the classification error is reduced slightly by about 0.1%. Compared to CondenseNetV2, the number of FLOPs is reduced by about 53%, the number of parameters is reduced by about 53%, and the classification error is reduced by about 0.78%. This proves the effectiveness of the multipath method and RSDC. We also experimented with the ImageNet dataset. Compared with CondenseNet, our proposed model (the bottom row of Table 5) reduces the number of FLOPs by more than 35% and improves top-1 classification error by more than 1.6%.

## 5. Discussion

We summarize the effect of our network as follows. First, the reuse of parameters is excellent. Unlike the existing convolutional neural network models that use only the last high-level feature maps and drop the previously produced feature maps, the DenseNet-based model uses both high-complexity feature maps as well as low-level feature maps to be more effective. Because the channel of DenseNet is narrow, it shows good performance with small parameters compared to other networks. Therefore, regularization is not required.

Second, our model satisfies the model complexity by crossing connections and passes one feature map to the other with a cross-shaped structure, not by increasing the number of channels. In addition, this mixing of information between paths has the same effect as shuffling for each group in ShuffleNet. The vanishing gradient problem is smoothed out by transferring the error directly to the beginning of the network during the backpropagation process.

Third, the numbers of FLOPs and parameters are reduced. In group convolution, the number of parameters decreases in proportion to the number of groups according to the relation:

$$HWk^{2}\frac{C}{G}\frac{M}{G}G=\frac{HWk^{2}CM}{G},$$

where *H* is the height of input feature maps, *W* is the width of input feature maps, *k* is the convolution filter size, *C* is the number of input channels, *M* is the number of output channels, and *G* is the number of groups.

Since our network is divided into *P* paths, the following should be satisfied:

$$HWk^{2}\frac{C}{PG}\frac{M}{PG}PG=\frac{HWk^{2}CM}{PG},$$

where *P* is the number of paths and the rest of the notation is the same as in the above equation. That is, our network has the effect of reducing parameters by 1/P compared to other models.

Finally, our network is divided by the number of paths defined in advance for each denseblock, which is more beneficial for parallelization than existing networks. Besides, our network can easily be plugged into any CNN that adopts the concatenation-based feature reuse mechanism.

However, our network takes about 4 times longer to train as compared to the original models (CondenseNet or CondenseNetV2). This is due to the following two reasons. First, we concatenate the output feature maps through the composite function from one path to the other. This process requires intensive computational resources while the original network does not due to the absence of the process. Second, the computational cost increases in the process of splitting the input feature maps and concatenating the output feature maps.

## 6. Conclusions

This paper has dealt with the effects of multiple paths and randomly selected dilated convolutions on lightweight deep networks. Our proposed network has multiple paths, and the diversity is enhanced by adding a ResNet in front of one path. We concatenate the output feature maps through the composite function from one path to the other. This helps to produce rich feature maps and is more suitable for parallelization than other models. The architecture of the proposed network is modularized and can be expanded by increasing or decreasing the number of paths. By adding RSDC instead of the 3×3 standard convolution, we obtain the effect of a large receptive field and improve the result. We compared our network with various SOTA networks and demonstrated better results (more than half the number of FLOPs and parameters, but similar classification error) on the CIFAR10/100 dataset.

In the future, we plan to apply multiple paths and RSDC to models other than DenseNet-based networks. Moreover, we need to train on datasets with larger input images, such as ImageNet [45]. It is expected that this will allow us to achieve meaningful results applying the large kernel size of RSDC.

## Figures and Tables

**Table 1:** Architecture of MLDN in CondenseNet/CondenseNetV2 on the CIFAR-10/100 dataset.

MLDN in CondenseNet	MLDN in CondenseNetV2	Feature Map Size
3×3 Conv (stride 1)	3×3 Conv (stride 1)	32×32×16
ResNet	ResNet	32×32×16
$\left[\begin{array}{cc} 1\times 1\;L-\mathrm{Conv} & \\ 3\times 3\;G-\mathrm{Conv} \end{array}\right]\times 14\times 2,k_{p}=4,\left(k=8\right)$	$\left[\begin{array}{cc} 1\times 1\;L-\mathrm{Conv} & \\ 3\times 3\;G-\mathrm{Conv} & \\ \mathrm{SFR}\;\mathrm{Module} \end{array}\right]\times 14\times 2,k_{p}=4,\left(k=8\right)$	32×32×128
2×2 average pool, stride 2	2×2 average pool, stride 2	16×16×128
$\left[\begin{array}{cc} 1\times 1\;L-\mathrm{Conv} & \\ 3\times 3\;G-\mathrm{Conv} \end{array}\right]\times 14\times 4,k_{p}=4,\left(k=16\right)$	$\left[\begin{array}{cc} 1\times 1\;L-\mathrm{Conv} & \\ 3\times 3\;G-\mathrm{Conv} & \\ \mathrm{SFR}\;\mathrm{Module} \end{array}\right]\times 14\times 4,k_{p}=4,\left(k=16\right)$	16×16×352
2×2 average pool, stride 2	2×2 average pool, stride 2	8×8×352
$\left[\begin{array}{cc} 1\times 1\;L-\mathrm{Conv} & \\ 3\times 3\;G-\mathrm{Conv} \end{array}\right]\times 14\times 8,k_{p}=4,\left(k=32\right)$	$\left[\begin{array}{cc} 1\times 1\;L-\mathrm{Conv} & \\ 3\times 3\;G-\mathrm{Conv} & \\ \mathrm{SFR}\;\mathrm{Module} \end{array}\right]\times 14\times 8,k_{p}=4,\left(k=32\right)$	8×8×800
8×8 global average pool	8×8 global average pool	1×1×800
FC softmax	FC softmax	

**Table 2:** Comparison of the classification error rates (%) of CondenseNet/CondenseNetV2 (original) and MLDN+CDN/MLDN+CDNV2 (proposed) on the CIFAR-10 and CIFAR-100 datasets.

Model	Paths (*P*)	Growth Rate (*k*)	FLOPs [M]	Params [M]	C-10	C-100
CDN		[8,16,32]	65.8	0.52	6.03	23.71
MLDN+CDN	[2,2,2]	[8,16,32]	34.9	0.28	5.94	22.96
	[2,4,4]	[8,16,32]	23.4	0.16	7.1	25.61
	[2,4,8]	[8,16,32]	20.3	0.12	8.86	27.2
MLDN+CDN with ResNet	[2,2,2]	[8,16,32]	35.6	0.28	5.9	22.76
	[2,4,4]	[8,16,32]	24.1	0.17	7.05	24.9
	[2,4,8]	[8,16,32]	21.1	0.12	7.7	26.86
	[2,2,2]	[16,16,16]	61.0	0.22	5.41	22.25
	[2,2,2]	[32,32,32]	231.4	0.78	4.75	21.58
	[2,2,2]	[16,32,64]	134.6	1.04	4.69	18.05
	[2,4,4]	[16,32,64]	88.8	0.72	4.93	19.71
	[2,4,8]	[16,32,64]	76.5	0.39	5.28	21.35
CDNV2		[8,16,32]	64.3	0.51	5.80	22.31
MLDN+CDNV2	[2,2,2]	[8,16,32]	33.4	0.27	5.66	21.34
	[2,4,4]	[8,16,32]	22.4	0.15	6.62	23.78
	[2,4,8]	[8,16,32]	20.0	0.10	8.54	25.71
MLDN+CDNV2 with ResNet	[2,2,2]	[8,16,32]	34.1	0.26	5.42	21.19
	[2,4,4]	[8,16,32]	23.2	0.16	6.48	23.18
	[2,4,8]	[8,16,32]	20.7	0.11	7.78	25.20
	[2,2,2]	[16,16,16]	59.9	0.20	5.15	20.50
	[2,2,2]	[32,32,32]	230.0	0.77	4.49	18.43
	[2,2,2]	[16,32,64]	133.1	1.04	3.66	16.37
	[2,4,4]	[16,32,64]	86.9	0.70	4.55	18.25
	[2,4,8]	[16,32,64]	74.5	0.38	4.83	19.70

**Table 3:** Comparison of the classification error rates (%) of CondenseNet/CondenseNetV2 (original) and MLDN+CDN/MLDN+CDNV2 and MLDN+RSDC+CDN/MLDN+RSDC+CDNV2 (proposed) on the CIFAR-10 and CIFAR-100 datasets.

Model	$k_{\mathrm{rsdc}}$	#L	C,G	FLOPs	Params	C-10	C-100
CDN		14	4	65.8M	0.52M	6.03	23.71
MLDN+CDN		14	4	35.6M	0.28M	5.9	22.76
MLDN+RSDC+CDN	5	14	4	36.1M	0.28M	5.5	21.88
	7	14	4	36.4M	0.28M	6.2	23.27
	5	14	2	66.6M	0.52M	6.37	28.68
	5	12	2	52.6M	0.41M	6.57	29.88
	5	10	2	40.3M	0.31M	6.84	30.66
	5	8	2	29.4M	0.22M	7.94	32.03
	5	6	2	20.1M	0.14M	8.92	34.24
CDNV2		14	4	64.3M	0.51M	5.80	23.01
MLDN+CDNV2		14	4	34.1M	0.27M	5.62	21.91
MLDN+RSDC+CDNV2	5	14	4	34.6M	0.27M	5.02	20.45
	7	14	4	35.2M	0.27M	5.88	22.30
	5	14	2	65.1M	0.5M	5.89	27.25
	5	12	2	51.7M	0.4M	6.00	28.18
	5	10	2	39.9M	0.29M	6.92	30.91
	5	8	2	28.2M	0.21M	7.68	31.24
	5	6	2	18.6M	0.13M	8.66	33.46

**Table 4:** Comparison of the classification error rate (%) with other convolutional networks on the CIFAR-10 and CIFAR-100 datasets.

Model	FLOPs	Params	CIFAR-10	CIFAR-100
ResNet-1001 [54]	2357M	16.1M	4.62	22.71
Stochastic-Depth-1202 [46]	2840M	19.4M	4.91	-
Wide-ResNet-28 [55]	5248M	36.5M	4.00	19.25
ResNeXt-29 [56]	10,704M	68.1M	3.58	17.31
DenseNet-190 [13]	9388M	25.6M	3.46	17.18
NASNet-A [40]	-	3.3M	3.41	-
CondenseNet(light)-160	1084M	3.1M	3.46	17.55
CondenseNet-182	513M	4.2M	3.76	18.47
ResNet-based				
CP [57]	62M	-	8.2	-
PFEC [58]	90M	0.73M	6.94	-
LECN [59]	124M	1.21M	5.27	23.91
NISP [60]	142M	0.96M	6.88	-
FPGM [61]	121M	-	6.24	-
DenseNet-based				
LECN [59]	190M	0.66M	5.19	25.28
CondenseNet [14]	66M	0.52M	6.03	23.71
CondenseNetV2-146 [15]	64M	0.51M	5.8	23.01
MLDN+RSDC-based				
MLDN+RSDC+CDN	36M	0.28M	5.5	21.8
MLDN+RSDC+CDNV2	35M	0.27M	5.02	20.45

**Table 5:** Comparison of classification error rate (%) with other SOTA networks on the ImageNet datasets.

Model	FLOPs	Params	Top-1	Top-5
Inception V1 [22]	1448M	6.6M	30.2	10.1
1.0 MobileNet-224 [35]	569M	4.2M	29.4	10.5
ShuffleNet 2x [36]	524M	5.3M	29.1	10.2
NASNet-A (N = 4) [40]	564M	5.3M	26.0	8.4
ShuffleNetV2 1.5x [38]	299M	-	27.4	9.4
1.0 MobileNetV2 [62]	300M	3.4M	28.0	9.0
MobileNetV3 L. 1.0x [39]	219M	5.4M	24.8	-
CondenseNet (G = C = 8) [14]	274M	2.9M	29.0	10.0
CondenseNetV2-C [15]	309M	6.1M	24.1	7.3
MLDN+RSDC+CDN	177M	2.3M	27.4	9.2

## Data Availability

CIFAR-10/100 dataset (accessed on 25 November 2021): https://www.cs.toronto.edu/~kriz/cifar.html, ILSVRC dataset (accessed on 25 November 2021): https://www.image-net.org/challenges/LSVRC/.

## Abbreviations

The following abbreviations are used in this manuscript:

CNN	Convolutional neural network
DCNN	Deep convolutional neural network
GPU	Graphics processing units
SOTA	State-of-the-art
MLDN	Multipath lightweight deep network
RSDC	Randomly selected dilated convolution
CDN	CondenseNet
CDNV2	CondenseNetV2
FLOPs	Floating point operations
Params	the number of parameters
FC	Fully connected layers
